# Supplementary material for: The causal role of circulating inflammatory markers in osteoporosis: a bidirectional Mendelian randomized study
Source: Front Immunol. 2024 Jul 18;15:1412298. doi: 10.3389/fimmu.2024.1412298 (PMC11291241; doi:10.3389/fimmu.2024.1412298)
Supplement: Supplementary file 1 [file DataSheet_1.zip › supplementary material/OSTEOPOROSIS-CIM/GCST90274811.tsv.gz_leave_one_out.pdf]

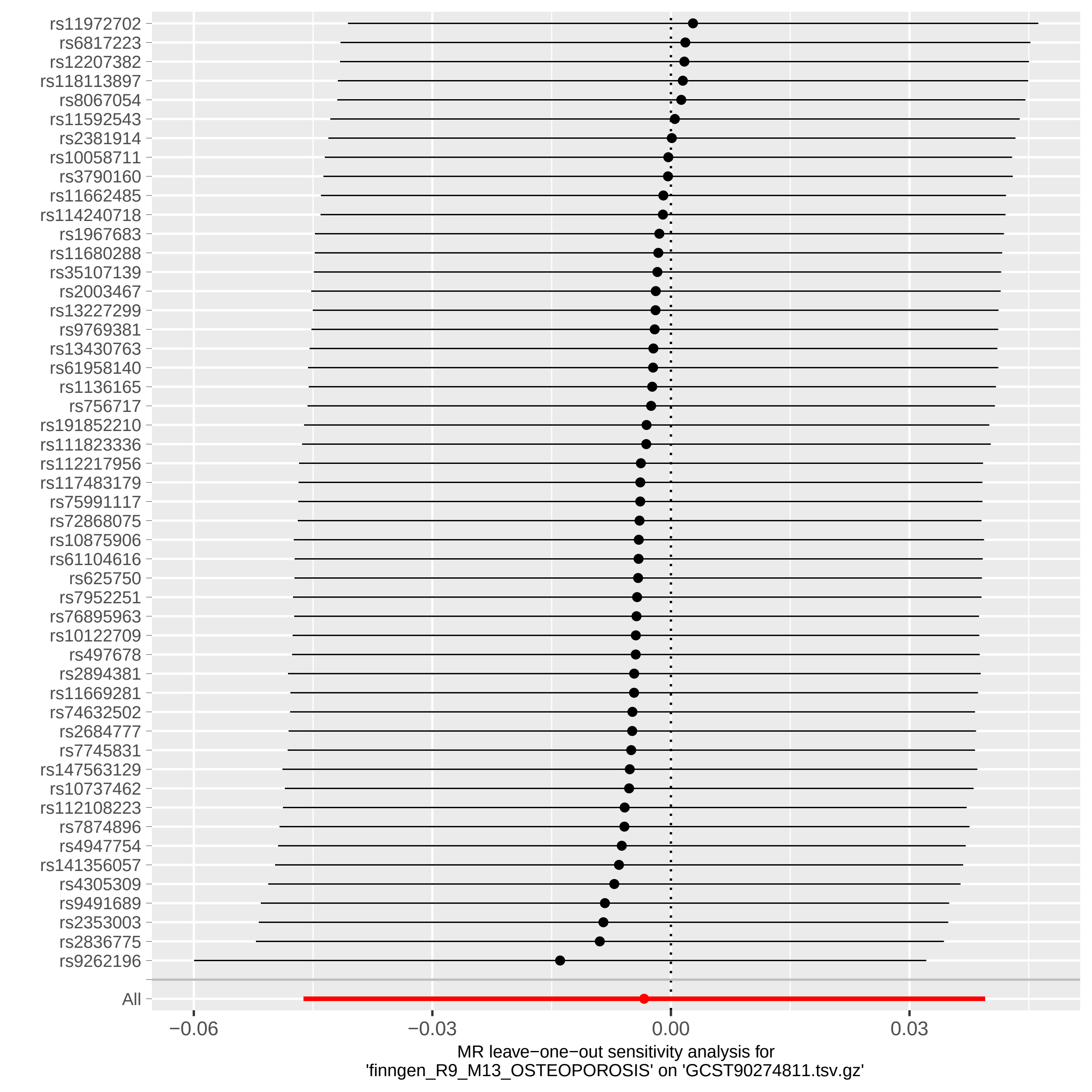
rs11972702
rs6817223
rs12207382
rs118113897
rs8067054
rs11592543
rs2381914
rs10058711
rs3790160
rs11662485
rs114240718
rs1967683
rs11680288
rs35107139
rs2003467
rs13227299
rs9769381
rs13430763
rs61958140
rs1136165
rs756717
rs191852210
rs111823336
rs112217956
rs117483179
rs75991117
rs72868075
rs10875906
rs61104616
rs625750
rs7952251
rs76895963
rs10122709
rs497678
rs2894381
rs11669281
rs74632502
rs2684777
rs7745831
rs147563129
rs10737462
rs112108223
rs7874896
rs4947754
rs141356057
rs4305309
rs9491689
rs2353003
rs2836775
rs9262196
All
−0.06
−0.03
0.00
0.03
MR leave−one−out sensitivity analysis for
'finngen_R9_M13_OSTEOPOROSIS' on 'GCST90274811.tsv.gz'
